# hsa_circ0021347 as a Potential Target Regulated by B7-H3 in Modulating the Malignant Characteristics of Osteosarcoma

**DOI:** 10.1155/2019/9301989

**Published:** 2019-12-17

**Authors:** Ling Wang, Guo-chuan Zhang, Fu-Biao Kang, Long Zhang, Ying-ze Zhang

**Affiliations:** ^1^Department of Orthopedics, The Third Hospital of Hebei Medical University, Shijiazhuang, Hebei, China; ^2^Department of Orthopedic Oncology, The Third Hospital of Hebei Medical University, Shijiazhuang, Hebei, China; ^3^Department of Liver Diseases, Bethune International Peace Hospital, Shijiazhuang, Hebei, China; ^4^Department of Orthopedics, The Second Hospital of Shanxi Medical University, Shanxi Key Laboratory of Bone and Soft Tissue Injury Repair, Taiyuan, China

## Abstract

In our previous study, we showed that B7-H3 played crucial roles in osteosarcoma (OS) development and might serve as a negative regulator of in osteoimmunology and help tumor cells escape immune surveillance. However, little is known about B7-H3 deficiency and its corresponding circRNA alteration or their relationship with osteosarcoma progression. Therefore, we established stable silencing of B7-H3 in OS cells and validated our results with western blotting and real-time PCR detection. Then, we performed a circRNA array to analyze the differential expression of circRNAs between the control and B7-H3 knockdown cells. The association between target circRNA expression and the clinicopathological features of patients with OS was further analyzed. As a result, hsa_circ0021347 was selected and validated to be significantly downregulated in OS tissues and cell lines and showed a strong negative relationship with B7-H3 expression in OS. In addition, clinicopathological features showed that hsa_circ0021347 in OS tissues was negatively associated with Enneking stage and positively associated with patients' survival. Finally, Gene Ontology (GO), Kyoto Encyclopedia of Genes and Genomes (KEGG), and PANTHER pathway analyses were performed to predict a network of hsa_circ0021347/miRNAs interactions to help us develop potential biomarkers for clinical diagnosis and design therapeutic strategies for OS.

## 1. Introduction

Osteosarcoma (OS) is the most common malignant type among bone tumors, with bimodal distribution with increased incidence around puberty [1, 2]. The rate of childhood and adolescent osteosarcoma ranged between 3 and 4.5 cases/million population/year [3, 4]. Epidemiological studies have revealed that the incidence of OS was closely correlated with skeletal growth, height, and disease appearance [5, 6]. However, the etiology of OS is still unclear, and thus, therapy is still focused on primary surgical resection and combined chemotherapy [7, 8]. In the current view of the OS milieu, nongenetic determinants, including the interaction of tumor cells and stroma, oxidative stress, and the immune system, also play crucial roles in cancer development. However, OS is unique to other solid tumors, for the bone and immune cells interact with each other and collaborate in the tumor microenvironment. Therefore, it might inspire us to find immunomodulatory molecule to design the appropriate therapy regimen.

B7-H3, also known CD276, is a type I membrane protein and shows a high similarity to the other B7 family members [9, 10]. B7-H3 has two different alternative isoforms containing repetitive IgV and IgC domains [11]. B7-H3 transcripts are universally expressed on both lymphoid and nonlymphoid organs, whereas its protein expression is limited to certain cell types, such as activated dendritic cells (DCs), monocytes, T cells, B cells, and NK cells [12, 13]. Many studies showed aberrant B7-H3 expression in a wide spectrum of cancers, including breast, lung, kidney, colon, liver, and prostate cancers and osteosarcoma [14–20].

In our previous study, we found evidence that B7-H3 expression was aberrantly present in osteosarcoma cells and tissues, contributing to tumor immune escape and invasive malignancy [20, 21]. In addition, enhanced sB7-H3 levels were found to be correlated with the clinical characteristics of OS patients and might be a potential biomarker associated with the pathogenesis of OS [22]. However, the in-depth regulatory mechanism of B7-H3 in OS remains elusive. CircRNAs could act as miRNA sponges to compete with endogenous RNAs in regulating posttranscriptional levels of gene expression. Therefore, in the present study, circRNA microarray and GO and KEGG pathway bioinformatics analyses were performed in B7-H3 knockdown (KD) OS cells to discover the biological functions of differentially expressed circRNAs and hypothetical B7-H3 downstream target genes.

## 2. Materials and Methods

### 2.1. Patients and Specimens

A total of 35 patients who were diagnosed with OS and subjected to primary surgical treatment in the Department of Orthopedics Oncology at the Third Hospital of Hebei Medical University from July 2016 to July 2018 were selected for the current study. Fresh paired tumor tissue and adjacent normal tissue samples were collected from primary tumors after surgical resection. The samples were evaluated and diagnosed as OS by two experienced pathologists independently. Written informed consent was obtained from all participants. The study was carried out under the ethical protocol approved by the Third Hospital of Hebei Medical University Ethical Committee.

### 2.2. Cell Culture and Treatment

The osteosarcoma MG-63 cell line was a kind gift from Dr. Zhi Lv, Shanxi Medical University, which was purchased from ATCC. Authentication of the original MG-63 cell line was provided by ATCC. The osteosarcoma cell lines were cultured in high-glucose Dulbecco's modified Eagle's medium supplemented with 10% fetal bovine serum, 100 U/mL penicillin, and 100 mg/mL streptomycin. All cells were maintained in a humidified atmosphere containing a 5% CO_2_ and 95% air atmosphere at 37°C. No ethical approval or informed consent was required to use the aforementioned cell line in this study.

### 2.3. B7-H3 Stable Knockdown Cell Line Construction

B7-H3-scienling recombinant lentiviral vector and negative control lentiviral vector were purchased from OriGen company, and the stable B7-H3-depleted OS cells were established according to the manufacturer's instructions by observing the expression of GFP. The set sequence of the B7-H3 shRNA contains 4 vials of gene-specific shRNAs in the pGFP-V-RS plasmid. We selected the most efficient one to carry out the following experiment. This sequence of the B7-H3 shRNA is 5′-TGAAACACTCTGACAGCAAAGAAGATGAT-3′. MG-63 cells were seeded into 96-well plates and allowed to grow at 70–80% confluence. Then, the cells were infected with retroviral particles (negative control) or B7-H3 shRNA in the presence of polybrene and incubated for 24 h at 37°C. At 48 h postinfection, the cells were cultured in the aforementioned medium containing puromycin (1 *μ*g/ml), and the medium was replenished every 2 days. After 7 days, stable cell lines were obtained and verified by qPCR and western blot analysis.

### 2.4. RNA Extraction and Real-Time PCR

Cells were extracted with TRIzol (Invitrogen, USA) and deposited in diethyl pyrocarbonate (DEPC) water. The miScript II RT Kit (Qiagen tec, China) was used to reverse transcribe RNA into cDNA with aliquots of 0.25 *μ*g of total RNA. Next, qPCR was performed using a SYBR Green qPCR Kit on the Applied Biosystems 7500 FAST Real-Time PCR System.

The small nuclear RNA RNU6B was used as an endogenous reference and purchased from Sangon Biotech. The primers for detecting hsa_circ0021347 and RNA RNU6B were as follows: sense: 5′-TCTGGACTCAGCCCTTTAC-3′ (forward), antisense: 5′- CAGTAGCTGCTCCCGTAA-3′ (reverse) for human hsa_circ0021347; 5′-TGCACCACCAACTGCTTAGC-3′ (forward) and 5′-GGCATGGACTGTGGTCATGAG-3′ (reverse) for human RNA RNU6B. The relative fold changes of candidate genes were analyzed using the 2^−ΔΔCT^ method.

### 2.5. Western Blot Analysis

Western blot analysis was performed to detect the effect of downregulation of B7-H3 in MG-63 cells 48 h after shRNA transfection. Cells were lysed in RIPA buffer (Sigma-Aldrich, USA) with complete protease inhibitor, and the protein concentration was determined by BCA assays. After routine denaturing, protein samples were loaded (25 *μ*g per well) and separated by 10% SDS-PAGE gel and then electrotransferred onto polyvinylidene fluoride membranes. The membranes were blocked in 5% skim milk at room temperature for 1 h. Primary antibodies, including B7-H3 and GAPDH (1 : 2000, Abcam), were incubated overnight at 4°C. Next, the membranes were washed with Tris-buffer and incubated with appropriate secondary antibodies according to the manufacturer's protocol. Finally, immunoreactive protein bands were visualized with an ECL Chemiluminescence kit (Pierce Biotechnology, Inc.).

### 2.6. Cell Invasion and Wound Healing Assays

For the cell invasion assay, B7-H3 KD cells and corresponding MG-63 control cells were seeded in a 24-well upper chamber coated with Matrigel membrane (BD Bioscience, USA) and cultured in serum-free DMEM medium overnight. Subsequently, 600 *μ*l complete medium with 10% FBS was added to the lower chambers as a chemoattractant. Cells were then incubated for 24 h at 37°C, and the invasive cells attached to the lower membrane surface were stained with 0.1% crystal violet, photographed, and counted.

For the wound healing assay, B7-H3 KD cells and control cells were used at a density of 5 × 10^5^ cells/well in six-well plates. The scratched wound was created using a pipette tip and rinsed twice with phosphate buffered saline to remove free floating cells and debris. Twenty-four hours after scratching, each group of cells began to migrate into the wound surface and was visualized to evaluate the average distance of the migrating cells. All experiments were repeated three times.

### 2.7. CircRNA Microarray Expression Profiling

The total RNAs extracted from the MG-63 control cells and KD cells were used for further circRNA microarray analysis. The concentration and purity of the RNA were measured by a NanoDrop ND-1000 instrument (Thermo Scientific, Waltham, MA, USA). The integrity of the RNA was evaluated using a Bioanalyzer 2100 (Agilent Technologies, Santa Clara, CA, USA). Then, Human circRNA array version 2.0 containing probes interrogating approximately 170,340 human circRNAs was used to detect the hybridized labelled RNAs. GeneSpring software V13.0 (Agilent Technologies, Santa Clara, CA, USA) was used to analyze the microarray data of the circRNAs. The threshold values of ≥2- and ≤−2-fold change and a *t*-test with *P* value of 0.05 were used to evaluate the significance of these differentially expressed circRNAs.

To elucidate the roles of differentially expressed circRNAs, Gene Ontology (GO) and KEGG (Kyoto Encyclopedia of Genes and Genomes) bioinformatics and data analyses were performed to annotate and predict the biological processes and molecular functions of selected circRNAs. Statistical significance of pathway correlations was determined by the enrichment score. The selected circRNA was verified to be differentially expressed in OS patients to further predict its target miRNAs and generate a circRNA-miRNA-mRNA predictive network map by Cytoscape3.5 software.

### 2.8. Statistical Analysis

All collected data are presented as the mean ± SEM and analyzed with SPSS 20 software (Abbott Laboratories, Chicago, IL, USA). The difference in circRNA expression was calculated by comparing expression levels between the B7-H3 KD group and the control cell group. Student's *t*-test was used to analyze the significance of these two groups. Significant results were regarded as fold change ≥2 and *P* values less than 0.05. A Pearson correlation test was used to evaluate the relationship between differential circRNAs and clinical parameters in OS patients. Kaplan-Meier curve analysis was used to predict the significance of the selected circRNA in predicting survival status.

## 3. Results

### 3.1. Generation of Stable Knockdown of B7-H3 Expression in OS Cell Clones

**Figure 1:**
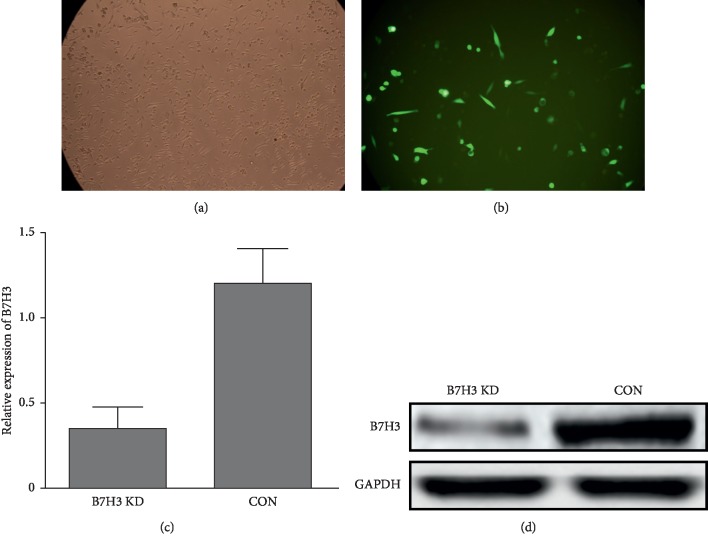
Stable B7-H3 knockdown MG-63 cells were generated via transfection of B7-H3 shRNA-expressing plasmid. (a) The morphology of the control group of MG-63 cells; (b) the morphology of MG-63 cells after B7-H3 shRNA transfection; (c) the B7-H3 mRNA expression level detected by qPCR; (d) the B7-H3 protein expression level detected by western blot analysis. KD: knockdown; CON: control group.

Our previous study showed that B7-H3 was overexpressed in OS tissues compared to benign bone disease, and B7-H3 expression was correlated with tumor stage, metastasis, and prognosis of OS patients [20, 22]. In the present study, we continued to explore whether B7-H3 was involved in OS cell invasion and its potential regulatory mechanism. The stable B7-H3 knockdown MG-63 cells were generated using shRNA transfection and grown in puromycin-containing medium. As shown in Figure 1, the efficacy and function of B7-H3 silencing in MG-63 cells were validated via qPCR and western blot assays compared to the scramble control. To further explore the biological role of reduced B7-H3 in MG-63 cell lines, transwell assays and wound healing assays were performed.

**Figure 2:**
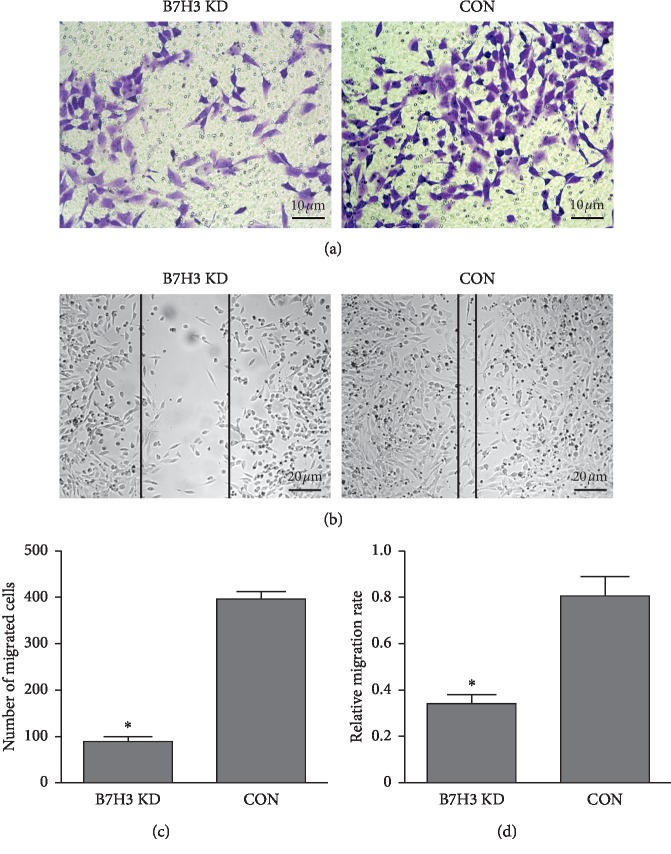
The invasive (a) and migratory ability of MG-63 cells before and after B7-H3 knockdown (b).

In the wound healing assay, scratch repair was significantly decreased in the MG-63 B7-H3 KD group, whereas it could be rapidly recovered in blank control cells. In the invasion assay, the average number of OS cells that passed through the Matrigel was significantly higher in the B7-H3 KD group than in the blank control group (Figure 2). Overall, the data strongly suggested that B7-H3 played important roles in regulating OS cell migration and invasion in vitro.

### 3.2. circRNA Expression Profiles Altered after B7-H3 Deletion in OS Cells

**Figure 3:**
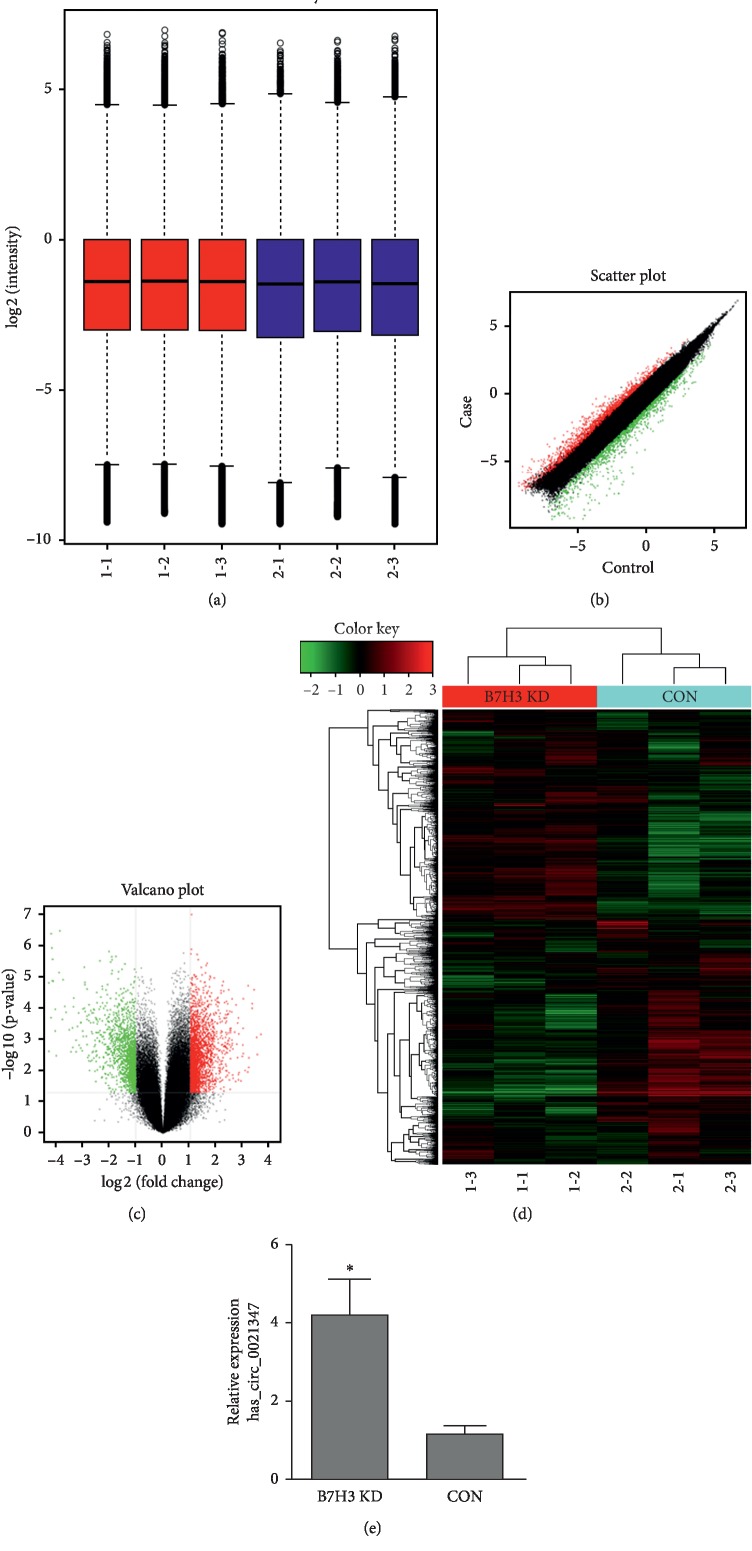
Differentially expressed circRNAs between B7-H3 knockdown MG-63 cells and control cells. (a) The distributions of circRNA intensities for the six compared samples are displayed as box plots after normalization. The scatter plot (b) and volcano plot (c) are displayed as expressed circRNAs between the B7-H3 knockdown and MG-63 cells. (d) Cluster heat map of the most differentially and significantly expressed circRNAs between the B7-H3 knockdown and MG-63 cells. (e) hsa_circ0021347 was validated by qPCR to determine the differential expression between the B7-H3 knockdown and MG-63 cells. Data represent the mean ± SD of three independent experiments; ^*∗*^*P* < 0.05 compared with the control.

Using a circRNA microarray assay, we identified 4526 differentially expressed circRNAs discriminating between human osteosarcoma MG-63 WT cells and B7-H3 KD cells. Box plots showed that the distributions of circRNA intensities for the six compared samples were nearly the same after normalization (Figure 3(a)). The differences in circRNA expression levels between WT and KD MG-63 cells are presented in a scatter plot. The green and red dots display the upregulated and downregulated circRNAs (FC ≥ 2.0), respectively (Figure 3(b)). In addition, a volcano plot was further used to indicate the significantly differentially expressed circRNAs in these two groups (FC ≥ 2.0, Figure 3(c)). Among these circRNAs, 652 circRNAs were differentially expressed, as shown by a *P* value of 0.05 and fold change of 2.0; 402 circRNAs were upregulated and 250 circRNAs were downregulated. After validation, hsa_circ0021347 was selected and predicted to be an effective potential target in OS cells after regulating B7-H3 (Figures 3(d) and 3(e)).

### 3.3. hsa_circ0021347 Might Be a Potential Target Regulated by B7-H3 in OS Cells

Recent studies have indicated that most frequently reported circRNAs play critical roles in binding and sequestering miRNA-mediated regulation of gene expression [23, 24]. To identify the miRNAs that bind to hsa_circ0021347, we performed a bioinformatics analysis to predict the circRNA-miRNA interaction via a multipredictive database. As a result, the 10 highest-ranking candidate miRNAs were identified (hsa-miR-646, hsa-miR-1205, hsa-miR-1286, hsa-miR-198, hsa-miR-383, hsa-miR-1206, hsa-miR-769-3p, hsa-miR-548g, hsa-miR-555, and hsa-miR-558), and 190 relative target mRNAs were predicted to have an interaction with hsa_circ0021347 in this study.

**Figure 4:**
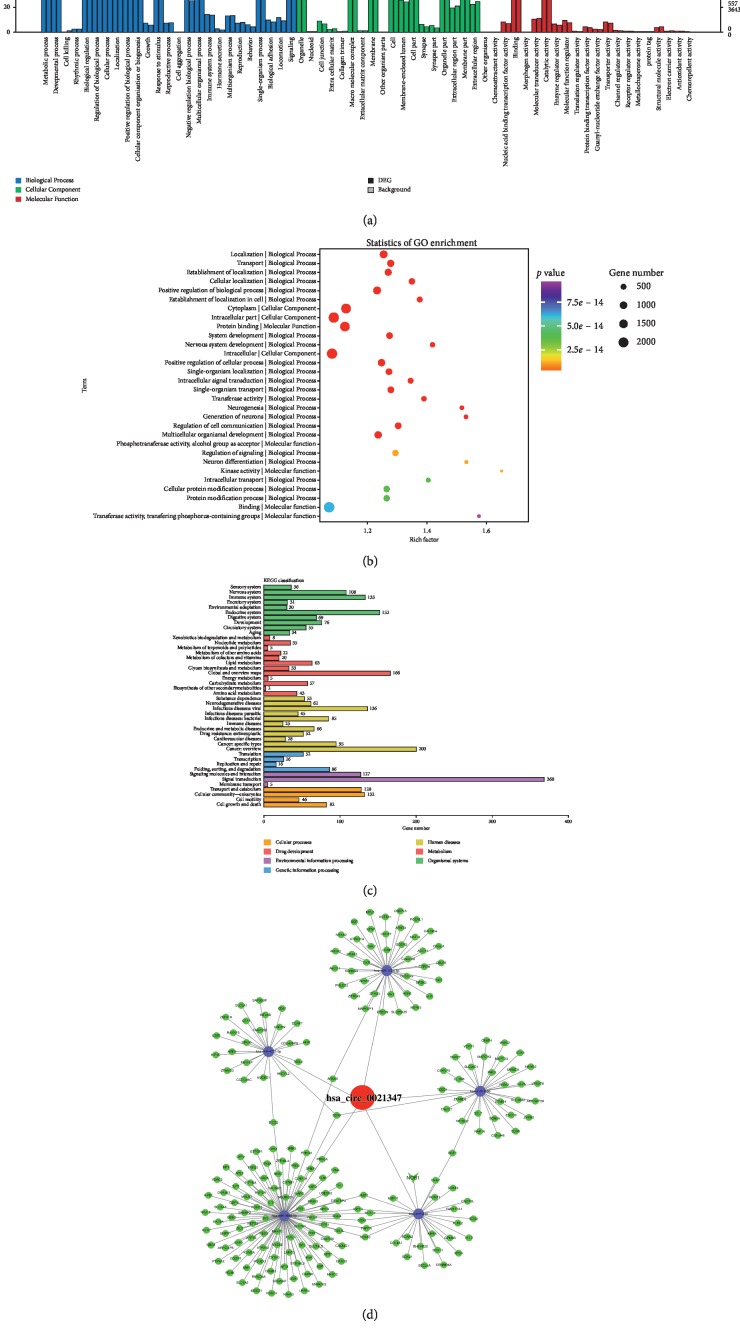
Functional annotations for target genes mediated by the hsa_circ0021347/miR-646 network via GO and KEGG analyses.

Among them, miR-646 and hsa-miR-1205 have been previously investigated and confirmed to be involved in suppressing or promoting the formation of OS [25, 26]. Other miRNAs, including hsa-miR-646, hsa-miR-1206, hsa-miR-198, hsa-miR-383, and hsa-miR-450, were previously confirmed to be closely related to different kinds of tumor development [27–30]. However, to the best of our knowledge, hsa-miR-1286, hsa-miR-555, and hsa-miR558 have never been researched. RNA-binding proteins (RBPs) can bind to circRNAs [31, 32]. Overall, the predictive results showed that the unique structure of hsa_circ0021347 might play crucial roles in recruiting downstream RNA or RBP complexes. According to the predicted results from miRanda, bioinformatics GO and PANTHER analyses were then performed and further elucidated the function of hsa_circ0021347 and its strong relationship with the cell localization, transport, and MAPK signaling pathways (Figure 4).

### 3.4. hsa_circ0021347 Was Downregulated in OS Tissue and Inversely Correlated with B7-H3 Expression

**Figure 5:**
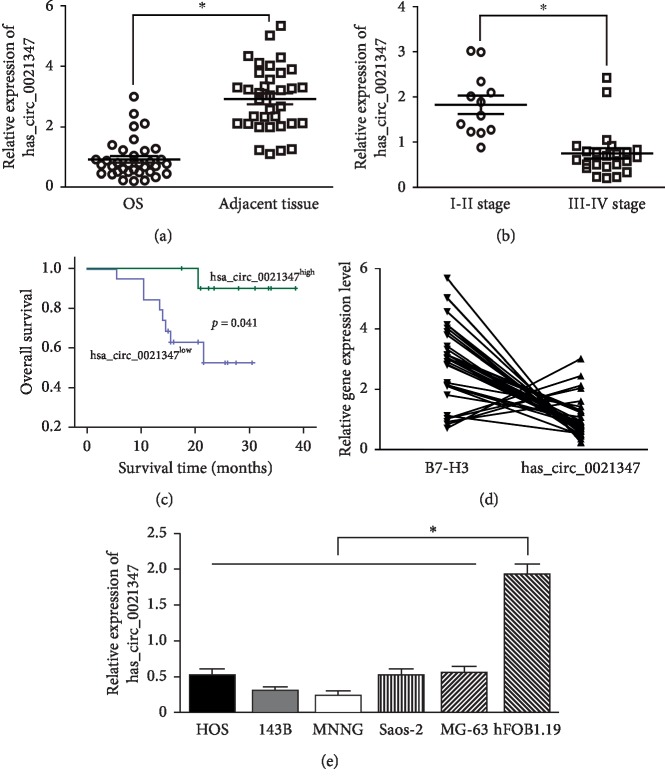
The expression levels of hsa_circ0021347 and its relationship with B7-H3 in OS tumor tissues and cells. (a) The expression levels of hsa_circ0021347 in tumor and adjacent normal tissues in OS patients. (b) The expression levels of hsa_circ0021347 in OS patients with different TNM stages. (c) ROC survival curve of hsa_circ0021347. (d) The relationship between hsa_circ0021347 expression and B7-H3 in OS. (e) The expression levels of hsa_circ0021347 in OS cells.

To illustrate the correlation between hsa_circ0021347 expression and clinical features in OS, 35 pairs of OS tissue samples and their corresponding adjacent normal tissues (connective tissue and muscles) were collected and detected via qRT-PCR (Figure 5(a)). According to statistics, abnormal expression of hsa_circ0021347 was not associated with age, gender, size, site, and differentiation in patients with OS. However, downregulation of hsa_circ0021347 was negatively associated with TNM stage (*P*=0.032, Figure 5(b)) but positively associated with patient survival (*P*=0.041, Figure 5(c)). In addition, the expression of hsa_circ0021347 was inversely related to the expression of B7-H3 (*r* = −0.757, *P*=0.018, Figure 5(d)). Similar to the results obtained from the clinical samples, we found that the expression of hsa_circ0021347 was markedly lower in several OS cell lines (HOS, 143B, MNNG, Saos-2, and MG-63) than in the osteoblast hFOB1.19 cell line. hsa_circ0021347 showed the comparatively lowest levels in HOS and 143B cells among these OS cells (Figure 5(e)).

## 4. Discussion

T-cell activation and effective cytotoxicity are crucial weapons against tumors; however, they are frequently frustrated because of the dysfunction of T-cell functions [33, 34]. As a general concept, the activation of antigen-specific T cells requires TCR recognition and costimulatory and coinhibitory molecules to deliver positive or negative signals [35, 36]. In 2001, B7-H3 was first discovered and cloned from a human dendritic cell cDNA library and showed 20%–27% amino acid sequence identity with other members of the B7 costimulatory family [10]. Research has demonstrated that B7-H3 accelerates tumor cell immune escape by inhibiting T-cell-mediated cellular immunity [37, 38]. In addition, B7-H3 has other nonimmunological regulatory functions in tumorigenesis and cancer development [39]. Existing evidence indicates that B7-H3 mRNA and protein levels fluctuated during the process of osteosarcoma and were especially overexpressed in the late stage [22, 40]. However, its potential regulatory mechanism has not been fully elucidated. Herein, we performed the following experiment to identify these mechanisms. The serum levels of interleukin 1 receptor antagonist (IL-1Ra), IL-6, and IL-8 were significantly increased in OS patients [41]. In our study, the inflammatory cytokine spectrum of OS cells changed significantly, especially secreted IL-1b, IL6, and IL-8.

CircRNAs, which were recently discovered in 1990, are a type of endogenous noncoding RNA and are stably and abundantly present in many species [42]. In this century, various vital physiological functions of circRNAs in humans have increasingly come to light, for example, acting as miRNA sponges [43, 44], transcriptional and translational regulators [45], regulation of gene expression [46, 47], and competing with linear splicing of pre-mRNAs [48, 49]. Accumulating evidence shows that many circRNAs are abnormally overexpressed in different tumor tissues, indicating that circRNAs could participate in carcinogenesis and tumor progression. Interestingly, some circRNAs exhibit a strong degree of tumor tissue specificity and are even associated with important clinical features, including tumor stage, grade, metastasis, and recurrence [50, 51]. Additionally, circRNAs in exosomes secreted by tumor cells can be transferred to normal cells, indicating that exo-circRNAs are important in the peritoneal metastasis of tumors [52].

Accumulating epidemiological data showed that the treatment for OS has remained essentially unchanged since the 1970s. The survival rates of OS patients were likewise maintained [53, 54]. A more intensive understanding of the mechanisms that drive metastasis and tumor heterogeneity coupled with available and clinically annotated data sets is urgently needed in the clinic. Recently, many studies have shown that an increasing number of circRNAs are aberrantly expressed in OS and perform crucial functions in the development of OS. In a previous study, hsa_circRNA_103801 and hsa_circRNA_104980 were shown to be highly expressed and involved in the initiation and development of OS, including the HIF-1, VEGF, and angiogenesis pathways [55]. Kun-Peng et al. also found that circPVT1 was meaningfully increased in OS tissues, serum, and chemoresistant cell lines, which showed a strong relationship with poor prognosis in OS patients. circPVT1 knockdown could deteriorate the chemoresistance of doxorubicin and cisplatin in OS cells via downregulating the expression of the classical drug resistance-related gene ABCB1 [56]. In our studies, the membrane and soluble B7-H3 forms were both highly expressed in OS tissues and cell lines and inversely correlated with the prognosis and recurrence of OS patients. Functional experiments showed that B7-H3 contributed to OS cell growth, invasion, and distant metastasis; however, its regulatory mechanism was unclear. Therefore, in this study, circRNA arrays were performed to identify the potential underlying transcriptional regulatory mechanisms. According to our data, 652 circRNAs were differentially present in B7-H3 knockdown MG-63 cells compared with the control group. Among these circRNAs, 402 circRNAs were upregulated, whereas 250 circRNAs were downregulated. Thus far, circRNAs could act as miRNA sponges to regulate downstream gene expression and thus interfere with relative RNA protein synthesis. circRNAs are usually enriched in miRNA-binding sites and can chelate miRNAs in an efficient way. The roles of circRNAs in OS may be related to miRNA-mediated effects. In fact, hsa_circ0021347 was identified in our study and was predicted to interact with over 200 miRNAs. Among these targeted miRNAs, miR-646 was a potential candidate that interacts with NOB1, which has shown potential efficacy in regulating tumor cell biology, especially invasion and metastasis.

In 2014, Li et al. reported that miR-646 could inhibit tumorigenesis of renal cancer cells through the modulation of NOB1 and thus influence the MAPK pathway in renal cancer [57]. In addition, a series of studies showed the importance of NOB1 in multitumor development, including papillary thyroid cancer [58], glioblastoma [59], gastric cancer [60], and osteosarcoma [61]. Recently, Liu et al. showed that the miR-646/NOB1 axis might play an important role in the development of OS and facilitate the colony formation, migration, and invasion ability of OS cells [26]. Therefore, we suggested that the hsa_circ0021347–miR-646-NOB1 axis may be involved in promoting tumor differentiation and invasion in osteosarcoma. However, further research is needed to validate this mechanism.

In summary, OS is one of the most predominant bone malignancies in minors, and B7-H3 knockdown could inhibit its tumorigenesis. After circRNA screening, hsa_circ0021347 was identified and significantly downregulated in OS cells and regulated by B7-H3. Although the number of samples analyzed in our study was relatively limited from a heterogeneous cohort of patients, the inverse relationship between hsa_circ0021347 and B7-H3 was identified and might be a promising target in OS in the near future. Bioinformatics analysis predicted that hsa_circ0021347-miR-646-NOB1 might serve as a novel pathway in OS development. However, the detailed molecular mechanism by which this circRNA contributes to OS proliferation, invasion, and metastasis requires further research.

## Data Availability

The datasets used and/or analyzed during the current study are available from the corresponding author on reasonable request.

## Supplementary Materials

Supplementary Materials Supplementary Figure 1: the proliferative (A), migratory (B-C), and invasive (D-E) abilities of MG-63 cells after upregulation of has_circ0021347. The figures of B-E are representative results shown at 48 h after upregulation of has_circ0021347. Each experiment has been replicated in triple. Supplementary Table 1: the clinical characteristics of the study patients' cohort of OS patients.
