# Supplementary material for: hsa_circ0021347 as a Potential Target Regulated by B7-H3 in Modulating the Malignant Characteristics of Osteosarcoma
Source: Biomed Res Int. 2019 Dec 17;2019:9301989. doi: 10.1155/2019/9301989 (PMC6948356; doi:10.1155/2019/9301989)
Supplement: Supplementary Materials — Supplementary Figure 1: the proliferative (A), migratory (B-C), and invasive (D-E) abilities of MG-63 cells after upregulation of has_circ0021347. The figures of B-E are representative results shown at 48 h after upregulation of has_circ0021347. Each experiment has been replicated in triple. Supplementary Table 1: the clinical characteristics of the study patients' cohort of OS patients. [file 9301989.f1.pdf]

Supple figure 1

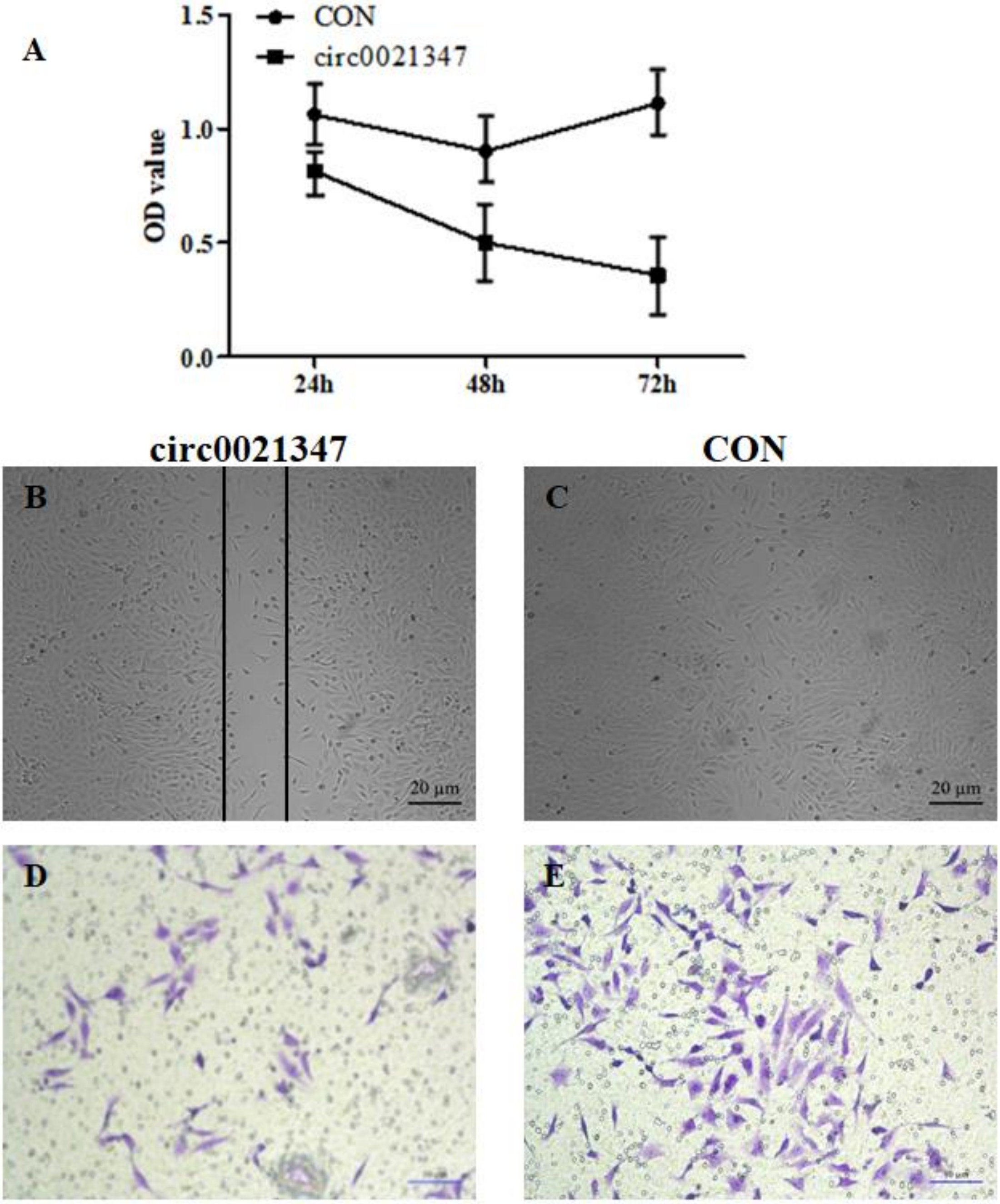

Supple table 1. Clinical characteristics of the study patients' cohort of OS patients

Variable	OS patients
Cases	35
Age	
≤10	20
>10	15
Gender	
Male	20
Female	15
Site	
Femur	17
Tibia	13
Others	5
Ennecking stage	
I-II stage	12
III-IV stage	23
Differentiation status	
High	20
Low	15
Lung metastasis	
Yes	10
No	25
